# Evidence for handheld electronic medical records in improving care: a systematic review

**DOI:** 10.1186/1472-6947-6-26

**Published:** 2006-06-20

**Authors:** Robert C Wu, Sharon E Straus

**Affiliations:** 1 Division of General Internal Medicine, University Health Network, Toronto, Ontario, Canada; 2 Knowledge Translation Program, University of Toronto, Toronto, Ontario, Canada

## Abstract

**Background:**

Handheld electronic medical records are expected to improve physician performance and patient care. To confirm this, we performed a systematic review of the evidence assessing the effects of handheld electronic medical records on clinical care.

**Methods:**

To conduct the systematic review, we searched MEDLINE, EMBASE, CINAHL, and the Cochrane library from 1966 through September 2005. We included randomized controlled trials that evaluated effects on practitioner performance or patient outcomes of handheld electronic medical records compared to either paper medical records or desktop electronic medical records. Two reviewers independently reviewed citations, assessed full text articles and abstracted data from the studies.

**Results:**

Two studies met our inclusion criteria. No other randomized controlled studies or non-randomized controlled trials were found that met our inclusion criteria. Both studies were methodologically strong. The studies examined changes in documentation in orthopedic patients with handheld electronic medical records compared to paper charts, and both found an increase in documentation. Other effects noted with handheld electronic medical records were an increase in time to document and an increase in wrong or redundant diagnoses.

**Conclusion:**

Handheld electronic medical records may improve documentation, but as yet, the number of studies is small and the data is restricted to one group of patients and a small group of practitioners. Further study is required to determine the benefits with handheld electronic medical records especially in assessing clinical outcomes.

## Background

Patient safety is an increasingly important issue in care delivery. The Institute of Medicine report estimates that 44,000 to 94,000 deaths are caused annually from medical errors [1]. Studies estimate that 3–17% of inpatients experience adverse events significant enough to prolong hospitalization, cause significant morbidity, or lead to death [2-10]. Errors can be caused by a number of factors including lack of information about the patient or lack of knowledge about a therapy. In a study of errors in medication prescribing, 30% were related to knowledge in drug therapy and 29% were due to a lack of patient information[11]. Decreased uptake of the evidence by the practitioner or patient can also cause errors of omission[12].

Handheld computers may improve quality of care by saving clinicians time in the accessing, retrieving and recording of data, allowing clinicians to focus more on patient care [13-15]. They can also provide clinical decision support at the point-of-care such as during electronic prescribing[16]. Improving access to knowledge databases at the point-of-care may also improve translation of knowledge into practice[17]. As well, many groups feel that mobile access to electronic medical records is the only way forward for certain complex care areas such as the emergency department[18,19].

Surveys estimate that approximately half of practicing physicians own a handheld computer[20,21]. Recent focus group sessions of 54 doctors from a variety of practice settings in the United States revealed that many use mobile computers in clinical practice, and some use them to access the electronic medical record[22]. They perceived the benefits to be improved productivity and accessibility of information as well as great potential to improve patient safety and quality of care. However, the true benefit of handheld computers is unclear and to help understand this issue, we conducted a systematic review of the evidence for mobile or handheld electronic medical records (EMRs) in improving patient care.

## Methods

### Definitions

For this paper, we used the American Health Information Management Association's definition of the *electronic medical record*: the computerization of health record content and associated processes usually referring to an electronic medical health record in a physician office setting or a computerized system of files[23]. Since we could not find a standard definition for a handheld EMR, we defined it as the computerization of health record content and associated processes available through a handheld computer, personal digital assistant (PDA) or tablet. Thus, for purposes of this study, handheld EMRs were not required to be integrated to or interfaced with a hospital medical record system.

### Search strategy

We searched MEDLINE, CINAHL, EMBASE, and the Cochrane Library from 1966 to September 2005 using the following search strategy: handheld technology AND electronic medical record AND randomized controlled trial. For handheld technology, the following terms were used: *computer peripherals*; *computers*, *handheld*; *handheld*; *mobile*; *pda*; *personal digital assistant*; *palm pilot*; *palmtop*; *point of care*; *tablet; *and *wireless*. The electronic medical record search used the following terms: *computer communication network*; *electronic chart*; *e-chart*; *epr*; *ehr*; *electronic health record*; *electronic patient record*; *hospital information systems*; and *medical records*. To identify randomized controlled trials (RCTs), we used the search strategy that has been developed and refined by the Cochrane Effective Practice and Organization of Care Group[24]. We retrieved potentially relevant articles and reviewed their reference lists for additional articles. The full search strategy is available from the authors upon request. There were no language restrictions.

### Inclusion criteria

Articles describing randomized trials or systematic reviews of randomized trials were included. We included studies if 1) we were able to extract relevant data, 2) they included an intervention group that used a handheld electronic medical record for patient care and a control group that was either a desktop EMR or the paper chart, 3) the users of the handheld EMR were clinicians, and 4) the outcomes had to be relevant to clinical care such as a decrease in errors, improved review of information, improved ordering of medications or tests, improved documentation or improved satisfaction.

### Outcomes

Two reviewers independently reviewed the search results and selected relevant publications that met the inclusion criteria. Disagreements were resolved by consensus. In cases of doubt, full text articles were retrieved for review and discussion. Full text articles of abstracts that met the inclusion criteria were retrieved. The investigators independently reviewed all full text articles to confirm that inclusion criteria were met. A standard data abstraction form was used to collect data from each article on the study design including study quality, participants, intervention, setting and relevant outcomes. Study quality was assessed using the following factors: blinding of participants or outcome assessors, concealed allocation, follow-up, and reliability of primary outcome measures. Differences in assessment by the reviewers were resolved through discussion.

Formal meta-analytic techniques including the pooling of data was not done due to the heterogeneous nature of the clinical interventions as well as the different outcome measures.

## Results

**Figure 1:**
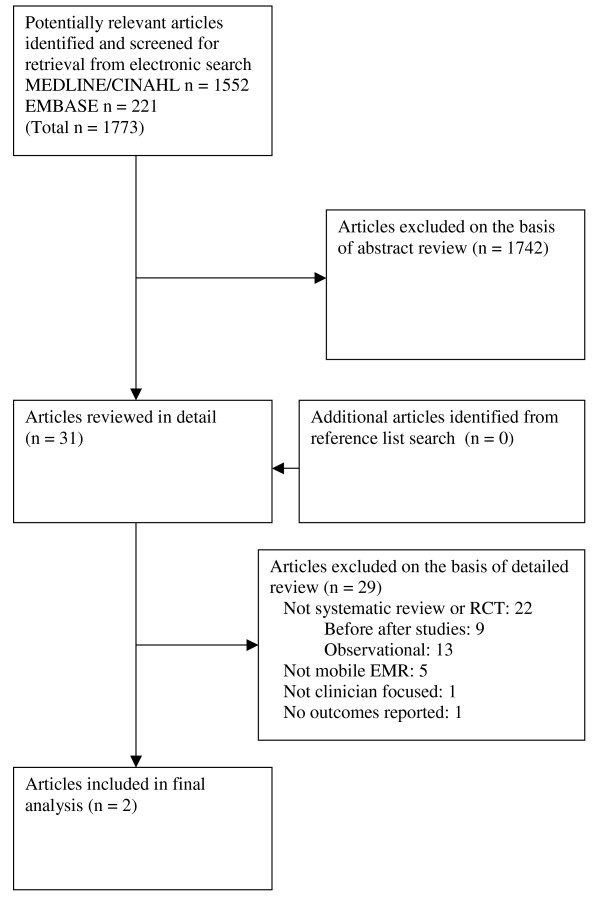
**Selection process for studies included in the analysis**. * RCT = randomized controlled trial; EMR = electronic medical record.

From 1773 citations that were screened, we retrieved 31 full text articles (Figure 1). Two articles met our criteria for inclusion (Table 1). The agreement between the 2 independent reviewers for article inclusion was excellent (kappa = 1.0). Reasons for excluding the articles were the following: 1) the study design was not a RCT (n = 22); 2) the intervention was not a mobile EMR (n = 5); 3) the intervention was clinician-focused (n = 1), and 4) there were no results reported (n = 1).

**Table 1:** Studies of handheld electronic medical records

**Study, Year**	**Setting**	**Patients**	**Practitioners**	**Primary Function**	**Results**	**Comments**
VanDenKerkof et al, 2004 [25]	Canada	Postoperative orthopedic patients (n = 72)	Anesthesiologists, n = 4	Documentation of acute pain management service	Encounter time 6.1 minutes in PDA group vs 4.6 minutes in paper chart (p = 0.00). Documentation significantly better for 3 of 5 main pain variables.	
Stengel et al, 2004 [26]	Germany	Orthopedic patients (n = 78)	Registrar (n = 1), residents (n = 3), and medical students (n = 2) in orthopedics	Documentation of daily progress, diagnoses	More diagnoses entered using PDA vs paper (411 vs 157).	More incorrect diagnoses using PDA vs paper (48 vs 7)

### Description of studies

The two studies examined 9 practitioners and 152 patients[25,26]. Both studies involved the care of orthopedic patients, and PDAs were compared to paper charts for purposes of documentation in both studies (Table 1).

VanDenKerkhof et al [25] looked at the use of PDAs by anesthesiologists working on a pain service and caring for orthopedic patients. Patients were randomized to have the anesthesiologist document their clinical assessment and place orders either on a PDA or on the paper chart as usual. The primary outcomes were the encounter time (defined as the time from reviewing patient information to assessing the patient and completing charting) and comprehensiveness of documentation. The encounter time was 6.1 minutes in the PDA group and 4.6 minutes in the paper chart group (p value = 0.00). Documentation comprehensiveness was determined by looking at five pain and side effect variables for each group. This was significantly better in the PDA group for three of the five variables including nausea, pruritus and sedation. For the two other variables, pain score and hypotension, there was a trend to increased documentation with the PDA group, but this was not statistically significant (p value 0.07 for both).

Stengel et al[26] studied 6 house officers providing care for orthopedic inpatients. Patients were randomized to have the house officers document diagnoses and clinical history for each patient using either the PDA or the standard paper form. The primary outcome was the number of documented ICD (*International Classification of Diseases*) diagnoses that were correct as determined by chart review. They found that more diagnoses were entered using the PDA (364 vs 150, P < 0.0001) after adjusting for false or redundant codes. Of note, there were 48 false or redundant codes documented in the PDA group compared with 7 in the control group.

### Methodological quality assessment

Both studies described the method of randomization and the study by Stengel documented concealed allocation. Blinding of outcome assessors was not done in either study due to the nature of the intervention and the choice of primary outcomes. Follow up was excellent in both studies. The reliability of primary outcome measures was difficult to determine. Neither used clinical outcomes but focused instead on comprehensiveness of documentation.

## Discussion

We identified 2 randomized trials that tested two different handheld mobile electronic medical records and both found improved documentation with use of handheld computers. In the study that measured documentation time, the group using PDAs took longer to document. In the study looking at number of diagnoses, the group using PDAs documented more correct diagnoses, but also recorded more redundant or false diagnoses.

There are several previous reviews of handheld applications in health care including reviews by Lu et al[13] and Fischer et al[27]. Both of these reviews provide a comprehensive picture of handheld adoption in healthcare and possible roles of PDAs. Lu characterizes current devices, benefits seen, adoption and complaints. Fischer describes specific uses from descriptive studies. Both articles summarize the literature to describe the functions that PDAs can perform as documented from a variety of study types including before-after and cohort studies. This research complements these papers by systematically reviewing the literature, using rigorous methodology to determine an estimate of the benefit from the highest quality evidence available.

A recent systematic review found that data collection by handheld computers is an effective alternative to paper methods[28]. There is some similarity between their systematic review and ours. Both review RCTs of handhelds, and both found studies that primarily assessed data collection or documentation. Yet the perspectives are different. Our review focused on the use of handheld electronic medical records, while their review included any form of handheld data collection. None of their included studies involved the use of handheld EMRs by clinicians. Instead, patients or healthy volunteers performed the data collection. Their review did find that data collection by handhelds was faster and preferred by users. The decreased handheld data collection time is different than what we found, but this is likely a result of different users and different applications.

There are several limitations to this study. The results are limited by the quality of studies included. Studies included different 'home-grown' handheld EMR systems so it is hard to generalize to other handheld EMRs. As well, both studies were in orthopedic patients. This clinical setting may be much more uniform and straightforward than other settings with greater variability such as the emergency department. As well, none of the studies looked at impact on clinical outcomes. Finally, studies had to be a RCT to be included in our review. However, it is important to note that there were no controlled trials excluded (Figure 1), minimizing the chance that a high quality study was missed. Less rigorous study designs such as before-after studies were not included.

The strength of this research is that it does synthesize what is currently known and it highlights areas for future research. More rigorous evaluations are required in multiple populations. Preferably, clinical outcomes should be measured. With our search, we found no primary or secondary outcomes evaluating changes in reviewing information, ordering by clinicians or improvement in patient care.

We note that neither study used wireless technology and instead used periodic synchronization. This may be due to wireless being a relatively newer technology. These RCTs were likely conceived years ago prior to widespread adoption of wireless technology. While wireless may have its benefits, it is unclear how well it will work in clinical practice.

## Conclusion

While handheld EMRs may improve patient care by improving documentation, reducing medical errors, and improving decision support, currently there is limited evidence of effectiveness. Further research is required in different populations and also focusing on improvement in patient outcomes. This highlights another area where informatics interventions are being implemented widely without rigorous evaluation.

## Competing interests

The author(s) declare that they have no competing interests.

## Authors' contributions

RCW and SES reviewed the literature search and abstracted information. RCW and SES wrote the manuscript. Both authors read and approved the final manuscript.

## Pre-publication history

The pre-publication history for this paper can be accessed here:


